# The link between thyroid autoimmunity (antithyroid peroxidase autoantibodies) with anxiety and mood disorders in the community: a field of interest for public health in the future

**DOI:** 10.1186/1471-244X-4-25

**Published:** 2004-08-18

**Authors:** Mauro Giovanni Carta, Andrea Loviselli, Maria Carolina Hardoy, Sergio Massa, Mariangela Cadeddu, Claudia Sardu, Bernardo Carpiniello, Liliana Dell'Osso, Stefano Mariotti

**Affiliations:** 1 Department of Public Health, Division of Psychiatry, University of Cagliari, Italy; 2 Department of Internal Medicine, University of Cagliari, Italy; 3 Department of Psychiatry, Neurobiology, Pharmacology, Biotechnology, University of Pisa, Italy; 4 Department of Public Health, University of Cagliari, Italy

## Abstract

**Background:**

To evaluate the association between mood and anxiety disorders and thyroid autoimmunity in a community sample. Methods: A community based sample of 222 subjects was examined. Psychiatric diagnoses were formulated using the International Composite Diagnostic Interview Simplified (CIDIS), according to DSM-IV criteria. All subjects underwent a complete thyroid evaluation including physical examination, thyroid echography and measure of serum free T4 (FT4), free T3 (FT3), thyroid-stimulating hormone (TSH) and anti-thyroid peroxidase autoantibodies (anti-TPO).

**Results:**

16.6% of the overall sample had an anti-TPO value above the normal cut-off. Subjects with at least one diagnosis of anxiety disorders (OR = 4.2, C.L. 95% 1.9–38.8) or mood disorders (OR = 2.9, Cl 95% 1.4–6.6, P < 0.011) were positive for serum anti-TPO more frequently than subjects without mood or anxiety disorders. A statistically significant association with anti-TPO+ was found in Anxiety Disorder Not Otherwise Specified (OR = 4.0, CL 95% 1.1–15.5), in Major Depressive Episode (OR = 2.7, CL 95% 1.1–6.7) and Depressive Disorder Not Otherwise Specified (OR = 4.4, S CL 95% 1–19.3).

**Conclusions:**

The study seems to suggest that individuals in the community with thyroid autoimmunity may be at high risk for mood and anxiety disorders. The psychiatric disorders and the autoimmune reaction seem to be rooted in a same (and not easy correctable) aberrancy in the immuno-endocrine system. Should our results be confirmed, the findings may be of great interest for future preventive and case finding projects.

## Background

Autoimmune thyroid disease may be linked to depression [1] and anxiety [2]. Autoimmune disease and depression are not uncommon: the prevalence of autoimmune thyroid disease in the community ranged from 4 to 25% [3] and lifetime prevalence of Major Depressive Disorder ranged from 6 to 17% [4]. Thus the association may have a great relevance in terms of public health and prevention.

The purpose of this investigation was to evaluate the relationship between mood and anxiety disorders and thyroid autoimmunity in a community survey. This research was carried out on the data base of two epidemiological studies aimed at defining the prevalence of psychiatric [5] and thyroid diseases [6] in Sardinia. On planning these surveys, researchers agreed to evaluate a representative sub-sample of a defined geographical area common to both endocrinological and psychiatric epidemiological surveys.

This paper present the results of the cross psychiatric and endocrinological evaluation from the common areas of the two surveys.

## Methods

The sample was extracted by randomization (1/10) subsequent to stratification according to age and sex, from the records of 2 Sardinian villages. Probands were interviewed face to face in their homes by specifically trained physicians. Two standardized forms were used to acquire information concerning: demographic data, state of health and use of social and health services. Psychiatric diagnosis was made using the Italian Simplified version of the Composite International Diagnostic Interview (CIDIS) [7]. The computer elaboration of data obtained enabled prevalence of psychiatric disorders according to DSM-IV [8] diagnostic criteria to be calculated.

Anti-thyroid peroxidase autoantibodies (anti-TPO), considered as the most sensitive and specific marker of thyroid autoimmunity [9] was determined by RIA (Sorin Biomedica Diagnostics, Saluggia, Italy) with a cut-off value of 20 IU/ml.

All subjects underwent a complete thyroid evaluation including physical examination, thyroid echography and measure of serum free T4 (FT4), free T3 (FT3), thyroid-stimulating hormone (TSH) and anti-thyroid peroxidase autoantibodies (anti-TPO). FT4 and FT3 were measured by means of a chromatographic method based on separation of free T4 on Lisophase columns (Technogenetics, Milan, Italy; normal values: FT4 6.6–16 pg/ml; FT3 2.8–5.6 pg/ml). TSH was measured by a chemiluminescent method (Ortho-Clinical Diagnostics Amersham, U.K.) with normal values ranging from 0.3–3.0 μU/ml. Thyroid echography was performed using a "real time" echograph (ALOKA Mod SSD 500 with a small parts 7.5).

The association of anti- TPO+ with the main diagnoses deriving from CIDIS interview was calculated using Odds Ratio. Statistical significance was calculated using the X^2^ test in 2 × 2 tables. Odds Ratio confidence intervals were calculated through application of the method of Miettinen [10].

Multivariate Logistic Regression was performed in order to evaluate the possible influences of gender and age on the association between anti-TPO+ and mood or anxiety disorders. The analysis was carried out considering mood (or anxiety) disorders as dependent variable, and anti-TPO+ (presence vs absence), gender (female vs male) and age (≤ 44 vs > 44) and their second order interactions as independent variables, by means of backward stepwise procedure; interactions lacking evidence of association (p > 0.20) were eliminated from the models.

## Results

From a total of 261 subjects identified (age >18 years), 222 (85.1%), 127 females (57.2%), and 95 males (42.7%); over 44 years 127 (57.2%), 79 females (62.2%,) 48 males (37.7%), agreed to take part in the study whilst 20 (8,7%) refused to participate and 19 (7.3%) could not be traced. The final sample did not differ respect to the population of origin with reference to the variables applied in stratification.

The lifetime prevalence of anxiety disorders in the sample was: Generalized Anxiety Disorder (GAD) 11.3%, Panic Disorder (PD) 2.7%, Anxiety Disorder Not Otherwise Specified (ADNOS) 5.4%, Social Phobia (SP) 5.4%; 18.5% had been diagnosed with at least one of these anxiety disorders. With regard to mood disorders, Major Depressive Episode (MDE) was present in 14.4%, Dysthymic Disorder (DD) in 2.7%, Depressive Disorder Not Otherwise Specified (DDNOS) in 4.0%; 18.9% had at least one of the above mentioned depressive disorders. 1.1% of the overall sample was affected by hypothyroidism, 16.6% had an anti-TPO value above the normal cut-off (anti-TPO+).

Table 1 shows the association between anti-TPO+ and lifetime psychiatric diagnosis. Subjects with at least one lifetime diagnosis of anxiety disorders or one lifetime diagnosis of mood disorders presented anti-TPO+ more frequently than subjects without mood or anxiety disorders. An association with anti-TPO+ was found in ADNOS, in MDE and DDNOS. The attributable risk for ADNOS was 0.54, 0.37 for MDE and 0.34 for DDNOS, other conditions as PD and DD presented very high attributable risk.

**Table 1:** Association between positivity anti-TPO (anti-TPO+), mood and anxiety diagnosis.

Diagnosis	Prev.nce N (%)	Anti-TPO+ N (%)	OR	IC 95%	χ2	P	Att.able Risk
One Anxiety Diagnosis GAD+PD+SP+ADNOS	41 (18.5)	15 (36.6)	4.2	1.9/38.8	12.6	0.001	0.54
One Mood Diagnosis MDE+DD+DDNOS	42 (18.9)	13 (30.9)	2.9	1.4/6.6	6.4	0.011	0.37
GAD	25 (11.3)	8 (32)	2.7	0.97/7.5	3.6	0.058	0.35
PD	6 (2.7)	3 (50)	5.4	0.7/37.3	2.8	0.096	0.68
SP	12 (5.4)	4 (40)	3.6	0.7/7.6	2.5	0.111	0.52
ADNOS	12 (5.4)	5 (41.7)	4.0	1.1/15.5	3.9	0.045	0.55
MDE	32 (14.4)	10 (31.2)	2.7	1.1/6.7	4.6	0.033	0.34
DD	6 (2.7)	2 (50)	5.2	0.3/16.8	1.3	0.250	0.67
DDNOS	9 (4.0)	4 (44.4)	4.4	1/19.3	3.8	0.049	0.60

GAD: Generalized Anxiety Disorder; PD: Panic Disorder; SP: Social Phobia; ADNOS: Anxiety Disorder Not Otherwise Specified; MDE: Major Depressive Episode; DD: Dysthymic Disorder; DDNOS: Depressive Disorder Not Otherwise Specified.

The Multivariate Logistic Regression showed that gender and age do not interact with anti-TPO either in mood (interaction anti-TPO-gender p = 0.97, OR 1.03, CI 95%, 0.19–5.48; interaction anti-TPO-age p = 0.62, OR = 1.52, CI 95%, 0.30–7.77), or in anxiety disorders (interaction anti-TPO-gender p = 0.93, OR = 0.93, CI 95%, 0.18–4.80; interaction anti-TPO-age p = 0.67, OR = 0.70, CI 95%, 0.14–3.64). Interactions were therefore eliminated from the models.

The final Logistic Regression models clearly indicated that gender and age do not influence the risk of one mood or anxiety diagnosis either as independent variables or as confounders (Table 2, Table 3 and Table 4).

**Table 2:** Frequency of anti-TPO+ in to the sample according sex and age and one mood diagnosis (OMD) and one anxiety diagnosis (OAD) diagnosis for multivariate logistic regression.

Age	Sex	N	anti-TPO+ (%)	OMD (%)	OMD (%) #anti-TPO+	OAD (%)	OAD (%) #anti-TPO+
<45	Female	79	17 (21.5)	17 (21.5)	6 (35.2)	13 (16.4)	6 (35.2)
<45	Male	48	8 (16.6)	7 (14.5)	2 (24)	7 (14.5)	3 (37.5)
>44	Female	48	7 (14.5)	10 (20.8)	3 (42.8)	14 (29.1)	4 (57.1)
>44	Male	47	5 (10.6)	8 (17.2)	2 (40)	7 (15.1)	2 (40)

#TPO+ in the sub-group is the total

**Table 3:** Multivariate logistic regression: effect of anti-TPO+ on risk of one mood diagnosis considering gender (female vs male) and age (≤ 44 vs > 44) effects.

	p	OR	CI 95%
anti-TPO+ vs anti-TPO-	0.01	2.89	1.31–6.38
Gender (F vs M)	0.37	1.38	0.68–2.82
Age (≤ 44 vs > 44)	0.71	1.14	0.57–2.30

**Table 4:** Multivariate Logistic Regression: effect of anti-TPO+ on risk of one anxiety diagnosis considering gender (Female vs Male) and age (≤ 44 vs > 44) effects.

	p	OR	CI 95%
anti-TPO+ vs anti-TPO-	0.001	4.50	2.02 – 10.04
Gender (F vs M)	0.23	1.58	0.75 – 3.31
Age (≤ 44 vs > 44)	0.08	1.91	0.92 – 3.96

## Discussion

The present study indicates an association between the presence of a lifetime diagnosis of mood or anxiety disorder and anti-TPO+ in a general population sample which had not been selected from medical or psychiatric health facilities. This association is independent by gender and age. Regarding specific diagnosis, MDE, DDNOS and ADNOS were associated with anti-TPO+.

This finding is consistent with several previous clinical studies providing evidence for a significant association of mood disorders or post-partum depression and symptomless autoimmune thyroiditis with or without sub-clinical hypothyroidism [11]. Association between hypothyroidism and mood disorders is however controversial as other authors maintain that bipolar disorders rather than unipolar depression are characterized by an increased risk for the presence of anti-thyroid antibodies [11]. However, a study performed by Fountoulakis and collaborators [1] recently found a link between autoimmune thyroid disease and Unipolar Depression. In this study, compared to control patients all depressive subtypes had significantly higher thyroid binding inhibitory immunoglobulins, and atypical patients had significantly higher thyroid microsomal antibodies. Thyroid function markers Free Triiodothyronine (FT3), Free Thyroxine (FT4), and Thyroid Stimulating Hormone (TSH) were normal in all subjects suggesting that Unipolar Depression might be characterized by a "low-thyroid function syndrome".

A sub-clinical dysfunction of axis Thyrotropin Releasing Hormone (TRH) – Thyroid Stimulating Hormone (TSH) with consequent alteration of circadian rhythms of TSH has been hypothesized in some depressive disorders. Indeed, this hypothesis may explain why some forms of mood disorders were associated with anti-TPO+ or thyroid autoimmunity without hypothyroidism, as defined by routine blood tests. A slight reduction in thyroid hormone secretion such as that found in sub-clinical hypothyroidism may affect cognition and mood [12]. At variance with other tissues which mainly rely on peripherally generated Triiodothyronine, the brain utilizes preferentially circulating thyroxine directly secreted by the thyroid gland and may become hypothyroid before other organs [13].

Moreover, a study carried out in a large community sample found no association between thyroid dysfunction, including hypothyroidism defined by thyroid blood test, and the presence of depression or anxiety symptoms [14]. This survey is rather limited due to the fact that the presence of depression or anxiety symptoms was defined using a self-rating scale, whilst in the present study depression and anxiety disorders were diagnosed by means of a structured psychiatric interview according to an international classifications. However, the link between thyroid autoimmunity and depression may involve other mechanisms related to the autoimmune pathogenesis of thyroid disease rather than hypothyroidism. Since several neuroendocrine secretory systems are involved in the control of immune reaction, a common neuroendocrine dysregulation involving cytokines might concur towards the pathogenesis of both affective disorders and autoimmune disease. Recent evidence suggests that thyroid autoimmunity may be affected by the Hypothalamic-Pituitary-Adrenal axis (HPA) through the balance of proinflammatory and antiinflammatory cytokines [15]. In line with this view, the increased frequency of post-partum depression, associated to the fact that pregnancy would seem to be a "protected" period, could explain at least in part the consequences on thyroid autoimmunity elicited by HPA-related modifications to the immunitary axis. Indeed, similar phenomena are observed in rheumatoid arthritis and multiple sclerosis [16,17].

With regard to the public heath aspects of this research, should these findings be confirmed, they would constitute a most important public health issue, due to the high attributable risk found. The attributable risk is a useful measure to document the burden of risk to a community. Attributable risk depends both on the magnitude of relative risk and on the prevalence of the risk factor in the population [4]. The high attributable risk of autoimmune thyroid for Major Depressive Disorder and anxiety and depressive sub-threshold syndromes may have implication for the development of preventive interventions. Particularly, further longitudinal studies will be required to confirm whether anxiety and depressive disorders are a consequence and not a cause of thyroid autoimmunity.

## Limitations

The potential of the study is reduced by the small sample size, particularly regard to psychiatric diagnoses less frequently observed in the general population, such as Panic Disorder; the extension of the findings is therefore rather limited.

## Conclusions

This study indicates an association between the presence of a lifetime diagnosis of mood or anxiety disorder and anti-TPO+. The psychiatric disorders and the autoimmune reaction seem to be rooted in a same (and not easy correctable) aberrancy in the immuno-endocrine system. If the findings are confirmed, they may prove to be of interest for future projects of case finding: a systematic screening for mood disorders in anti-TPO+ subjects and a systematic evaluation for thyroid diseases and thyroid autoimmunity in subjects with mood disorders may be advisable.

## Competing interests

None declared.

## Authors' contributions

MGC conceived the study, participated in the design of the study, performed the statistical analysis and drafted the manuscript. AL, SM and CS participated in the statistical analysis and drafted the manuscript. MCH, SM, MC, BC, LDO participated in its design and coordination. All authors have read and approved the final manuscript.

## Pre-publication history

The pre-publication history for this paper can be accessed here:


